# From self-organization in relativistic electron bunches to coherent synchrotron light: observation using a photonic time-stretch digitizer

**DOI:** 10.1038/s41598-019-45024-2

**Published:** 2019-07-17

**Authors:** Serge Bielawski, Edmund Blomley, Miriam Brosi, Erik Bründermann, Eva Burkard, Clément Evain, Stefan Funkner, Nicole Hiller, Michael J. Nasse, Gudrun Niehues, Eléonore Roussel, Manuel Schedler, Patrik Schönfeldt, Johannes L. Steinmann, Christophe Szwaj, Sophie Walther, Anke-Susanne Müller

**Affiliations:** 1 Univ. Lille, CNRS, UMR 8523 - PhLAM - Physique des Lasers, Atomes et Molécules, Centre d’Étude Recherches et Applications (CERLA), F-59000 Lille, France; 2 Karlsruhe Institute of Technology (KIT), D-76131 Karlsruhe, Germany; 3 Present Address: Fraunhofer Institute of Optronics, System Technologies and Image Exploitation (IOSB), D-76275 Ettlingen, Germany; 4 Present Address: Paul Scherrer Institute (PSI), 5232 Villigen, Switzerland; 5 Present Address: DLR (Deutsches Zentrum für Luft und Raumfahrt) Institute of Networked Energy Systems, Carl-von-Ossietzky-Str. 15, D-26129 Oldenburg, Germany; 6 Present Address: DESY (Deutsches Elektronen-Synchrotron), Notkestr. 85, D-22607 Hamburg, Germany

**Keywords:** Imaging and sensing, Experimental particle physics, Terahertz optics, Photonic devices, Ultrafast photonics

## Abstract

In recent and future synchrotron radiation facilities, relativistic electron bunches with increasingly high charge density are needed for producing brilliant light at various wavelengths, from X-rays to terahertz. In such conditions, interaction of electron bunches with their own emitted electromagnetic fields leads to instabilities and spontaneous formation of complex spatial structures. Understanding these instabilities is therefore key in most electron accelerators. However, investigations suffer from the lack of non-destructive recording tools for electron bunch shapes. In storage rings, most studies thus focus on the resulting emitted radiation. Here, we present measurements of the electric field in the immediate vicinity of the electron bunch in a storage ring, over many turns. For recording the ultrafast electric field, we designed a photonic time-stretch analog-to-digital converter with terasamples/second acquisition rate. We could thus observe the predicted link between spontaneous pattern formation and giant bursts of coherent synchrotron radiation in a storage ring.

## Introduction

**Figure 1:**
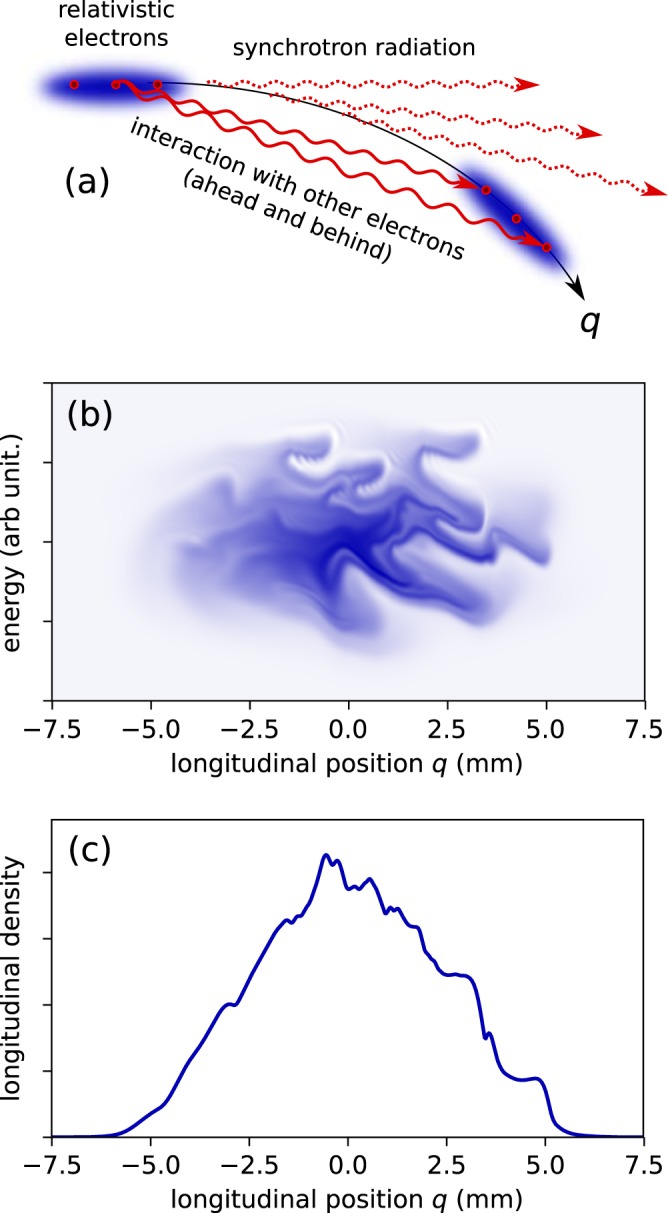
Microbunching instability in a relativistic electron bunch. (**a**) Illustration: at accelerator locations where curved trajectories are present, each electron interacts with the coherent synchrotron radiation emitted by the others. (**b**,**c**) Numerical simulation: resulting spontaneous appearance of a pattern in phase space, which evolves in a complex way (computation using KARA storage ring parameters, see Method Section, and Supplementary Video 1). (**c**) Corresponding longitudinal density profile. Note that the fast modulation in (**c**) – thought apparently small – is responsible for a particularly intense emission of coherent synchrotron radiation (typically 10^3^–10^5^ times the normal synchrotron radiation) ranging from the millimeter-wave to THz domains.

Current storage ring synchrotron radiation facilities involve challenges in photonics, both for understanding the light source and for realizing suitable ultrafast measurement devices. Generation of light for users is performed by using electron bunches in the subnanosecond to picosecond range, with high charge density. This density is so high that the light emitted by the electrons affects the dynamics of neighboring electrons in a dramatic way. In particular, this nonlinear collective effect leads to spontaneous formation of small-scale structures (in the sub-millimeter to centimeter range) in the longitudinal profile of electron bunches^1–17^. This is known as the *microbunching instability*^3,4,18,19^ (see Fig. 1). This effect is conceptually close to the universal mechanisms of pattern formation in Nature^20^ due to interaction between parts of the same system, such as the modulation instability in optical fibers^21^, sand ripple formation induced by the wind or under the sea^22^, or phantom traffic jams^23^.

However, besides being a fascinating phenomenon of light and matter self-organization, latest generation light sources must systematically consider these collective effects for very practical reasons. Spontaneous formation of small-scale microstructures can have a deleterious effect on electron bunch stability and emission properties, and they are at the same time a tremendous source of coherent radiation in the terahertz domain^3–17^, provided the instability can be mastered. This is the reason why understanding and controlling the interplay between Coherent Synchrotron Radiation (CSR) and the microbunching instability has nowadays become a central open question in the development of synchrotron radiation facilities.

To answer this question, it is essential to develop ultrafast photonic devices for electron bunch shape characterization. The challenges for the photonics community is high, given the need for ultrashort (picosecond or femtosecond) temporal resolution, single-shot operation, at high repetition rates (MHz and more), and given the particularly challenging environment near relativistic electron bunches. Recent advances consequently pushed photonics systems beyond the state of the art. Ultrafast electric-field measurement techniques using femtosecond laser pulses (electro-optic sampling^24^) have allowed single-shot bunch shape measurements (plural)^25^, and these techniques have then been extensively investigated and improved this last decade^26–30^. As these techniques require compact femtosecond lasers, this also motivated specific work on fiber-based sources, using parabolic pulse amplification^31,32^. This even led to new record spectral widths for parabolic pulse amplifiers^33^.

Ultrafast diagnostics also recently started to use strategies from the emerging field of “photonic hardware accelerators”^34^, which aims at increasing the speed of electronic devices by combining them with specially designed photonic front-ends. In particular *photonic time-stretch analog to digital converters*^35,36^ opened the way to the realization of “single-shot terahertz oscilloscopes”^30,35,37–39^ providing up to tens of million traces per second.

The availability of new ultrafast measurement systems led to several milestones in these storage ring investigations. Pioneer experiments using a streak camera could visualize microstructures in the several GHz range at the VUV ring^40^. More recently, electron bunch shapes have been indirectly characterized in single-shot by using new detectors based on thin films of superconducting YBCO^41^, and high repetition rate electro-optic sampling, using photonic time-stretch^37^. Although this progress enabled to record structures in single-shot up to the THz range, the obtained information concerned only the far-field (i.e., the synchrotron radiation) emitted by the microstructures^37,39,40^.

In this article, we present a photonic system that enables to observe microstructures and their evolution in a direct way, by monitoring the electric field in the immediate vicinity of the electrons.

## Results

### Experimental strategy

**Figure 2:**
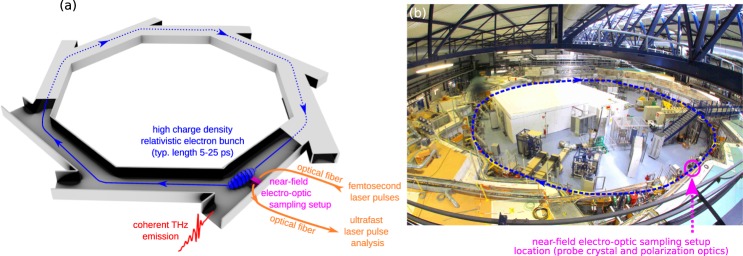
Global strategy of the experiment (**a**), and picture of the KARA storage ring (**b**). Interaction of a relativistic electron bunch with its own emitted coherent radiation leads to the so-called microbunching instability, and formation of a pattern with few millimeter period in the longitudinal direction. For monitoring the longitudinal electron bunch shape, we record the electric field evolution in its vicinity (at few millimeters), using a specially designed picosecond-speed *photonic-time-stretch analog-to-digital converter*. The digitization is made in two steps: (i) laser pulses are modulated by the electric field using an electro-optic crystal, and (ii) the modulated pulses are analyzed in single-shot, picosecond resolution, and multi-MHz acquisition rate. Note that the crystal is actually placed above the electron beam (the whole photonic time-stretch digitizer is detailed in Fig. 3). The electron bunch microstructure is also emitting intense coherent synchrotron radiation (CSR), which is simultaneously recorded. KARA photograph by Carina Franck (licensed under CC BY).

Recording bunch shapes in a non-destructive way required two open problems to be solved. The first one consisted in probing the electric field by approaching an electro-optic crystal at few millimeters from the relativistic electron bunch (Fig. 2), without losing the electron bunch or damaging the crystal. By carefully designing the experimental setup^42,43^, we could demonstrate the possibility to operate the synchrotron facility with an electro-optic crystal at 2–18 millimeters from the electron bunch. This pioneer experiment at the ANKA (now KArlsruhe Research Accelerator – KARA) storage ring thus opened the way to real-time investigations of storage ring electron bunch shapes, under the condition that a suitable photonic ultrafast readout system can be designed.

Optical readout of the crystal birefringence versus time was the second key problem as measurements had to be be performed: (i) in single-shot, (ii) with picosecond or sub-picosecond resolution, (iii) at several MHz acquisition rate. Moreover this ultrafast readout needs to be performed with an important dynamical range because the fast microstructures are expected to appear as a small modulation superimposed on a large slowly-varying background (see Fig. 1c). At KARA, we have been exploring two directions in parallel. We have been developing a new generation of fast linear cameras (KALYPSO) with multi-MHz acquisition rates^44–47^. In parallel, we have been developing a second direction consisting in using the so-called photonic time-stretch strategy^35,36^. The latter strategy allows up to tens of MHz acquisition rate, using an association of commercial detectors and electronics. The results presented in this article are obtained with this strategy.

### Photonic time-stretch analog-to-digital converter

**Figure 3:**
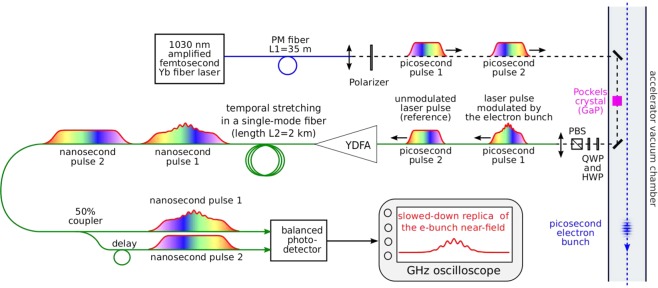
Photonic time-stretch analog-to-digital converter realized for recording the shape of electron bunches at high repetition rate. The electron bunch near-field is imprinted onto a chirped laser pulse, by using the Pockels effect in a gallium phosphide (GaP) crystal. The laser pulse is then further chirped in a long fiber, so that the modulation is slowed down to the nanosecond range, and can be recorded by an oscilloscope. Furthermore, an additional laser pulse which has not interacted with the electron bunch is used as “zero field” reference, and is subtracted from the signal by a balanced photodetector. Note that another reference laser pulse (not shown) is also recorded and used in the offline data processing (see Methods and Supplementary Material). Blue line: polarization-maintaining (PM) fiber, green lines: single-mode non- polarization maintaining (SM) fibers. YDFA: ytterbium-doped fiber amplifer, HWP: half-wave plate, QWP: quarter-wave plate, PBS: polarizing beam splitter. The GaP crystal is placed above the electron bunch trajectory. Only the free-space optics (along the dashed line) is located near/in the vacuum chamber, the rest (laser source, YDFA and downstream components) is located in a remote laboratory.

The photonic time-stretch digitizer setup is represented in Fig. 3. The optical front-end combines two parts. A single-shot electro-optic sampling (EOS) system^42^ imprints the electric field shape onto a chirped laser pulse^24,25^. Then, the laser pulse exiting the EOS system is stretched in a 2 km dispersive fiber, so that the picosecond information is temporally stretched to the nanosecond range, and can be recorded using a photodetector and a conventional oscilloscope (5 GHz bandwidth is typically used here, see Methods). If we start from a compressed laser pulse, the output signal should be a replica of the ultrafast electric pulse, slowed-down by a factor^35,36^:

1 $$M\,\mathrm{=1}\,+\frac{{L}_{2}}{{L}_{1}},$$

with *L*_1_ and *L*_2_ the fiber lengths before and after the crystal (if the fibers are identical). Since an unknown amount of extra-dispersion is also present before the fiber of length *L*_1_, we also measured the stretch factor experimentally. We found *M* = 75.8, i.e., 1 nanosecond on the oscilloscope corresponds to a real duration of 13.2 ps at the input for all results presented hereafter.

In order to increase the signal-to-noise ratio (and thus the dynamic range), we combined the amplified photonic time-stretch strategy^48^, with a balanced detection technique. The signal is amplified using a home-made ytterbium-doped fiber amplifier before entering the 2 km fiber. Moreover, at each electron bunch passage, three laser pulses are sent into the system (only one of the pulses being modulated by the electron bunch). The modulated pulse and a reference pulse are subtracted at the analog level, using a balanced photodetector (see Fig. 3). Furthermore, the second reference pulse allows a dark reference to be available at the data analysis stage (see Methods section and supplementary material).

### Simultaneous measurement of electron bunch shapes and resulting coherent radiation emission

**Figure 4:**
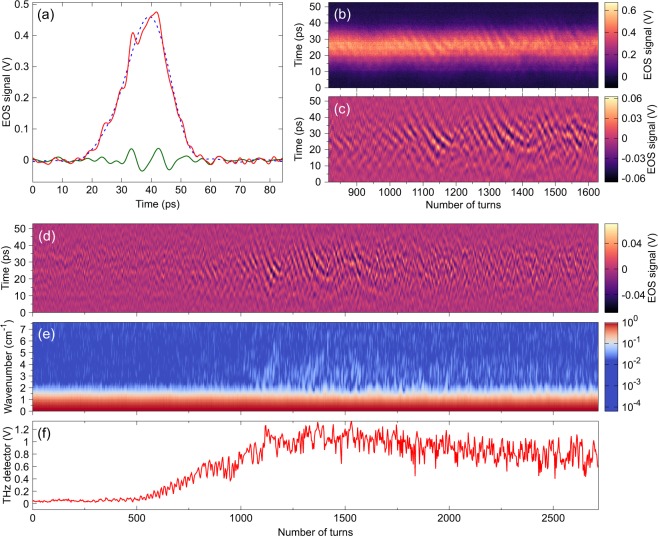
Simultaneous recording of the electron bunch shape at each turn and associated emission of coherent synchrotron radiation (CSR). (**a**) Single-shot recording of an electron bunch shape that passes near the detection electro-optic crystal (electric near-field recorded using time-stretch electro-optic sampling). Red: electro-optic signal (over the 0–250 GHz bandwidth). Green: high frequency part between 90 and 250 GHz. Blue: low frequency part below 80 GHz. (b,c,d) Single-shot bunch shapes versus turns in the storage ring: (**b**) Total electro-optic sampling signal (unfiltered), (**c**) and (**d**): high frequency part (90–250 GHz) revealing the microbunching structure [(**c**) is a zoom of (**d**)]. (**e**) Power spectrum of each bunch shape versus turn number (the colormap has been normalized with respect to the global maximum). (**f**) Emitted coherent synchrotron radiation recorded simultaneously at the KARA infrared beamline using a THz diode detector (the pulse height is represented at each turn). Note the correlation between the increase in coherent synchrotron radiation emission in (**f**) and the spontaneous formation of microstructures (**d**,**e**).

A typical single-shot electro-optic signal is represented in Fig. 4a. The data correspond to the longitudinal density profile of the electron bunch (or more precisely to the electric field in its vicinity, see Methods and supplementary material). Detailed analysis reveals two components. As expected, a slowly-varying shape is systematically observed, whose width is of the order of the electron bunch size. When the electron bunch is “compressed” below a threshold size, a fast modulation appears on the electro-optic sampling signal. Technically, this was obtained by decreasing the momentum compaction factor α of the storage ring, i.e., using the so-called low-alpha operation.

In order to conclude non-ambiguously that this structure corresponds to the microbunching instability, we represented the data as a function of the revolution number (Fig. 4b–d). High-pass filtered data reveal that the rapidly evolving structure occurs in bursts (Fig. 4c), and their space-time evolutions (Fig. 4b) present a characteristic pattern. As we will see, this will be a central point for comparing data with theory.

Since the electro-optic sampling is performed at each turn in the storage ring, it is possible to examine the correlation of the spontaneous microstructure formation, with the appearance of coherent synchrotron radiation. In Fig. 4f we have plotted the data over a long time range, together with the signal synchronously recorded with a millimeter-wave diode detector placed at our infrared beamline. We can clearly see the correlation between the occurence of a burst of CSR, and the growth of the microstructure. This correlation was systematically observed in the recorded data.

## Discussion

These new data sets can be compared to existing models of electron bunch dynamics. The physics of the electron bunch evolution involves essentially three ingredients: (i) acceleration and energy losses at each turn, (ii) interaction of each electron with the field created by the whole electron bunch distribution, (iii) and the relation between their energy and the revolution time in the storage-ring. The evolution equation for the distribution of the electrons in phase space may be written in the form of a Vlasov-Fokker-Planck equation^18,19^:

2 $$\frac{\partial f}{\partial \theta }-p\frac{\partial f}{\partial q}+[q-{I}_{c}{E}_{wf}(f,q)]\frac{\partial f}{\partial p}\mathrm{=2}\varepsilon \frac{\partial }{\partial p}(pf+\frac{\partial f}{\partial p}),$$

where $f(q,p,\theta )$ is the distribution of the electrons at time *θ* in phase space (*q*, *p*). *θ* is a continuous and dimensionless variable associated to the number of turns in the storage ring: $\theta =2\pi {f}_{s}t$, where *t* is the time (in seconds) and *f*_*s*_ is the synchrotron frequency (here in the tens of kilohertz range). The longitudinal position *q* and relative momentum *p* are the deviation from the so-called synchronous electron (with position *z*_0_ and energy *E*_0_). *q* and *p* are expressed in units of the equilibrium bunch length ${\sigma }_{z}$ and energy spread ${\sigma }_{E}$ at zero current. ${I}_{c}{E}_{wf}(f,q)$ corresponds to the field created by the whole bunch at the location *q*. We use here only shielded CSR impedance. Details are given in the Methods section.

**Figure 5:**
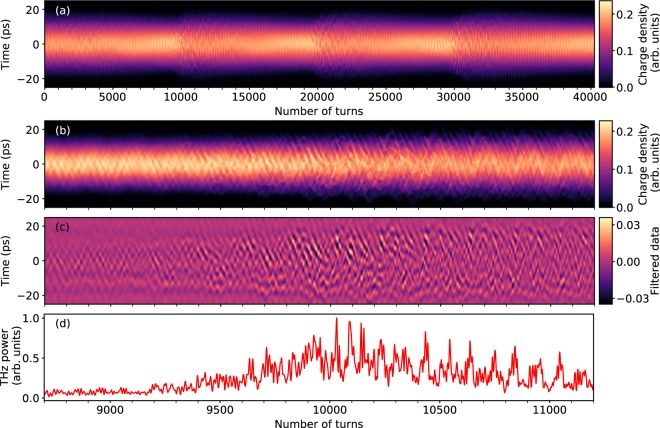
Numerical simulation of the electron bunch dynamics. (**a**,**b**) Electron bunch shape at each turn in the storage ring **(b)** is a zoomed view of one of the bursts of **(a)**. **(c)** Filtered data (in the 90–250 GHz range), revealing the microstructure evolution. **(d)** Coherent synchrotron radiation emitted by the microstructure. See Fig. 1 for the associated longitudinal density profile and phase-space at turn 9742, and Supplementary Video 1 for the corresponding phase space evolution.

In Fig. 5, we have represented the simulated evolution of the electron bunch shape versus number of turns in the storage ring. We can see that this type of representation can be used directly for performing in depth tests of theoretical model versus experimental data. In our case, we can see that the model can reproduce part of the spatio-temporal features, as e.g., structures moving towards the bunch head, and bunch tail. Evolution versus number of turns also reveals interesting discrepancies between model predictions and experimental data. These types of measurements should allow in due course to refine the models of the wakefields created by each electron (whose Fourier transform is known as the *machine impedance*).

In conclusion, we present a strategy enabling a simultaneous measurement of the “shape” of electron bunches in a non-destructive manner at each turn in a storage ring, by monitoring their electric fields. This new measurement possibility enables to directly observe the correlation, at each turn, between the charge modulation and the underlying coherent synchrotron radiation emission, and was predicted for storage rings more than a decade ago^18,19^. This type of strategy will enable to start very stringent tests of theoretical models of relativistic electron bunch dynamics, that were not possible before. We believe that this direct access to the microbuching instability and coherent synchrotron radiation properties may provide an important milestone on the way to master the instabilities, either for suppressing them, or make them usable as a stable source of THz radiation.

In a general way, electron bunch shape diagnostics is expected to address challenging questions to the photonic community. An important (and related) open question concerns the non-destructive characterization of electric field oscillations, when the structures are in the few to tens of microns range (and thus the time scales are in the few femtosecond range). This is an important question for studies of microbunching instabilities in lastest generation free-electron lasers, and would require to perform single-shot electro-optic sampling of mid-infrared pulses. This may represent one of the next milestones in the development of photonic systems destined to relativistic electron bunch characterization.

## Methods

### Laser system

The 1030 nm probe pulses are produced by a mode-locked ytterbium-doped fiber laser, operating at 62.5 MHz, and synchronized to the RF reference of the KARA storage ring. An acousto-optic pulse picker selects 3 pulses per turn in the storage ring. The pulses are compressed and then amplified in a polarization-maintaining ytterbium-doped fiber parabolic pulse amplifier^43^. The output pulses have a typical bandwidth of 80 nm FWHM.

### Near-field electro-optic sampling setup

The laser pulses are then transported in a 35 m-long polarization maintaining fiber to the electro-optic measurement system installed in the storage ring. Thanks to a Treacy compressor placed before the fiber, output pulses can be adjusted in the few tens of ps range. The electro-optic sampling is performed by an 5 mm-long GaP crystal placed inside the vacuum chamber, above the electron beam. The crystal can be moved towards the electron beam orbit and was placed – for the data shown – at a distance of $\approx 4$ mm from the electron bunch. The quarter-wave plate and half-wave plate (see Fig. 3) are adjusted so that the system is operated near extinction^29,49^, in order to obtain high sensitivity. The light exiting the low-power port of the beam-splitter is injected in a single-mode fiber and transported back for analysis in the remote photonic analysis station (placed outside of the storage ring shielding).

### Amplified photonic time stretch system

The modulated chirped laser pulses are first amplified using a home-made ytterbium-doped fiber preamplifier, and then stretched by propagation in a 2 km-long single-mode fiber (Corning HI 1060). The fiber’s output is then split using a thin-film 3 dB splitter (see Fig. 3), and the delay between output ports is exactly one repetition period of the mode-locked laser (16 ns). Thus a reference (i.e., unmodulated) laser pulse is subtracted from the laser pulse which carries the ultrafast modulation using the balanced photodetector. The balanced photodetector is an InGaAs amplified photoreceiever (DSC-R412 from Discovery Semiconductors), with a 20 GHz bandwidth. The photoreceiver specifications for gain and noise are 2800 V/W and 40 pW/$\sqrt{{\rm{Hz}}}$ (both being specified at 1550 nm). The precise delay and relative power levels between the two photodetector inputs are adjusted using an adjustable delay line and a variable optical attenuator. Data are recorded using a Lecroy Labmaster 10 Zi oscilloscope (with an – overdimensioned – 30 GHz bandwidth and 80 Gs/s acquisition rate), and the acquired data are numerically low-passed filtered at 5 GHz before signal analysis (corresponding to 380 GHz at the electro-optic crystal location).

Each recorded pulse is a replica of the electric field in the near-field of the electron bunch, which is “stretched in time” by a factor $M=75.8$. In other words, 1 ns at oscilloscope input corresponds to 13.2 ps at the electro-optic crystal. The oscilloscope’s 80 gigasamples/s acquisition rate corresponds to an effective sampling rate of 6.06 terasamples/s. The post-processing filtering to 5 GHz corresponds to an input analog bandwidth limitation of 380 GHz.

### Data processing

At each turn in the storage ring, three consecutive pulses are emitted by the laser, and only the last one interacts with the electron bunch near-field. Thus, at turn *n*, the balanced detector signal contains four pulses: (i) the raw balanced EOS signal ${V}_{n}^{EOS}(t)$, (ii) a reference balanced signal without EOS modulation ${V}_{n}^{REF}(t)$, and two saturated pulses corresponding to unbalanced pulses (see supplementary Figs 1–2). The EOS signal represented here corresponds to ${V}_{n}^{EOS}(t)-{V}_{n}^{REF}(t)$. The spectra of the EOS signal (as in Fig. 4e) show that a reasonable signal-to-noise ratio is observed up to 5 GHz bandwidth (i.e., 380 GHz at input). Hence raw data were first low-pass filtered at 5 GHz, before data analysis (i.e., a 5 GHz oscilloscope would be sufficient for the present recording). Then we proceeded to further filtering for examining the different parts of the spectra. In particular, high frequency structures of Fig. 4c,d (and the green curve in Fig. 4a) are obtained by filtering the data in the 1.19 GHz-3.30 GHz band (i.e., 90–250 GHz at the input). Unfiltered data are represented in Fig. 4b.

The electro-optic sampling signals (as represented in Fig. 4a–e) hence represent the electric field evolution, multiplied by the laser pulse shape, (see Supplementary material for the signal details).

### Coherent sychrotron radiation analysis

The THz pulses are detected at KARA’s IR1 infrared beamline, using an amplified 140–220 GHz Schotty barrier diode detectector (Virginia Diodes Inc. WR5.1ZBD) connected to a 6 GHz oscilloscope (Lecroy SDA760ZI-A). Figure 4e represents the recorded detector pulse height versus revolution number.

### Accelerator parameters

The results presented in this article are performed in single bunch operation, at $E=1.287$ GeV energy, for a current $I=1.625$ mA, an acceleration voltage of 1500 kV and a momentum compaction factor of $\alpha =0.724\times {10}^{-3}$. The storage ring revolution frequency is 2.716 MHz.

### Numerical simulations

Numerical simulations have been performed using the semi-Lagrangian scheme^50^, and the shielded CSR wakefield as in refs^37,41^. Calculations have been made and cross-checked using two independently developed codes. One code is a parallel implementation of the Warnock scheme^50^ using MPI (Message Passing Interface), and the other code is INOVESA which has been developed by the KIT group^51^. Figure 5 is provided by the first code, parameters are summarized in the Supplementary material.

## Supplementary information

Supplementary material

Supplementary Video 1

## Data Availability

The data that support the findings of this study are available from the corresponding author upon reasonable request.
