# Supplementary material for: From self-organization in relativistic electron bunches to coherent synchrotron light: observation using a photonic time-stretch digitizer
Source: Sci Rep. 2019 Jul 17;9:10391. doi: 10.1038/s41598-019-45024-2 (PMC6637151; doi:10.1038/s41598-019-45024-2)
Supplement: Supplementary file 1 — Supplementary material [file 41598_2019_45024_MOESM1_ESM.pdf]

From self-organization in relativistic electron bunches to coherent synchrotron light: observation using a photonic time-stretch digitizer

Serge Bielawski[1,*], Edmund Blomley[2], Miriam Brosi[2], Erik Bründermann[2], Eva Burkard[1,3], Clément Evain[1], Stefan Funkner[2], Nicole Hiller[2,4], Michael J. Nasse[2], Gudrun Niehues[2], Eléonore Roussel[1], Manuel Schedler[2], Patrik Schönfeldt[2,5], Johannes L. Steinmann[2], Christophe Szwaj[1], Sophie Walther[2,6], and Anke-Susanne Müller[2]

[1] *Univ. Lille, CNRS, UMR 8523 - PhLAM - Physique des Lasers, Atomes et Molécules, Centre d'Étude Recherches et Applications (CERLA), F-59000 Lille, France.*

[2] *Karlsruhe Institute of Technology (KIT), D-76131 Karlsruhe, Germany*

[3] *Present address Fraunhofer Institute of Optronics, System Technologies and Image Exploitation (IOSB), D-76275 Ettlingen, Germany*

[4] *Present address Paul Scherrer Institute (PSI), 5232 Villigen, Switzerland.*

[5] *DLR (Deutsches Zentrum für Luft und Raumfahrt) Institute of Networked Energy Systems, Carl-von-Ossietzky-Str. 15, D-26129 Oldenburg, Germany*

[6] *Present address DESY (Deutsches Elektronen-Synchrotron), Notkestr. 85, D-22607 Hamburg, Germany*[*]

(Dated: February 16, 2019)

I. SIMULATION PARAMETERS

Parameters are displayed in Supplementary Table I

Physical machine parameters	
Energy	1.285 GeV
Current	1.625 mA
Momentum compaction factor α	0.724×10^{-3}
Revolution frequency	2.71579 MHz
Bending magnet radius of curvature	5.559 m
Vacuum chamber height	32 mm
Zero current RMS bunch length σ_z	1.21428 mm
Relative RMS energy spread δ	0.47×10^{-3}
Synchrotron damping time	10.4 ms
Synchrotron frequency (νs)	13.5 kHz
**Numerical parameters used for the simulations**	
Integration mesh size	$20\sigma_z \times 20\delta$
Number of mesh points	896 points × 896 points
Time step	$\frac{1}{4000} \times \frac{1}{\nu_s}$
Number of CPU cores	64

Table I. Parameters corresponding to experimental results and used in the numerical simulation.

II. RECORDED OPTICAL SIGNALS: TIMING AND TYPICAL RAW DATA

At each electron bunch turn, 3 laser pulses are sent to the electro-optic sampling setup:

- Two reference pulses that do not interact with the electron bunch electric field (one only being displayed in the article's Figure 3, for clarity purposes).
- One pulse that interacts with the electron bunch electric field.

The timing of the signals is schematically represented in supplementary Figure 1. The balanced detector's output contains two useful informations:

- The electro-optic (EO) signal.
- A background reference corresponding to "zero electric field".

If the two balanced port were perfectly symmetric as displayed in the figure, this background should be zero. Actually small asymmetries are present, hence the first step of the analysis consists of subtracting the EO and background signals.

A typical experimental raw signal (with nonzero background and EO) is represented in Figure 2.

* Corresponding author : serge.bielawski@univ-lille.fr

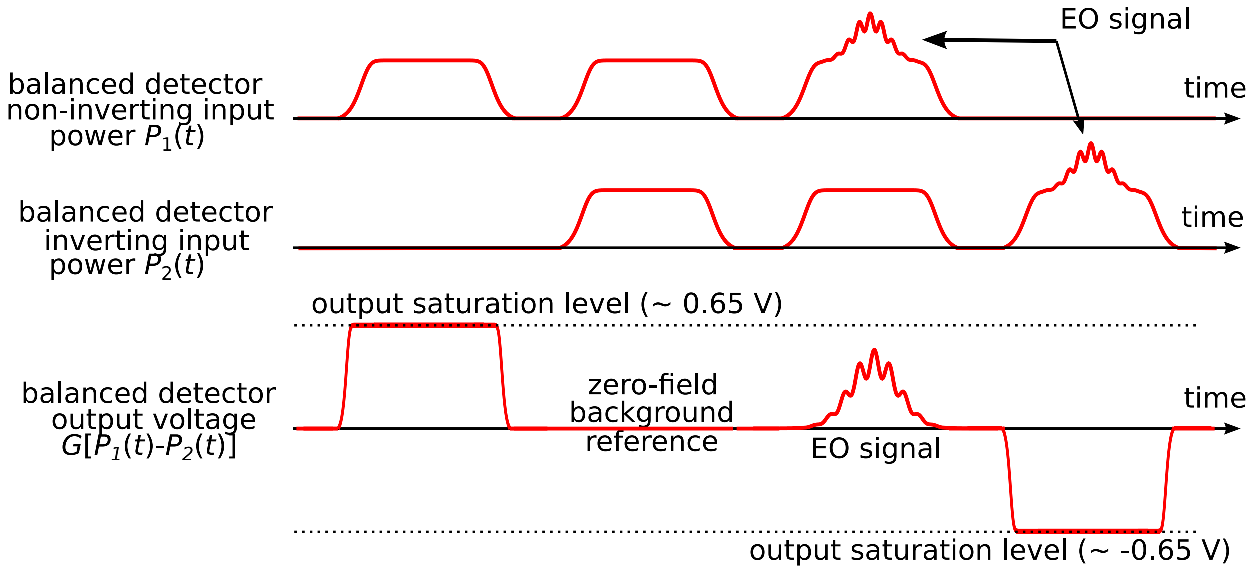

Figure 1. Timing of the signals (illustration) at the balanced photodetector's inputs and output. The two useful output signals are: the electro-optic (EO) signal, and the "zero-field" background. Note that the EO signals at input and output are not to scale.

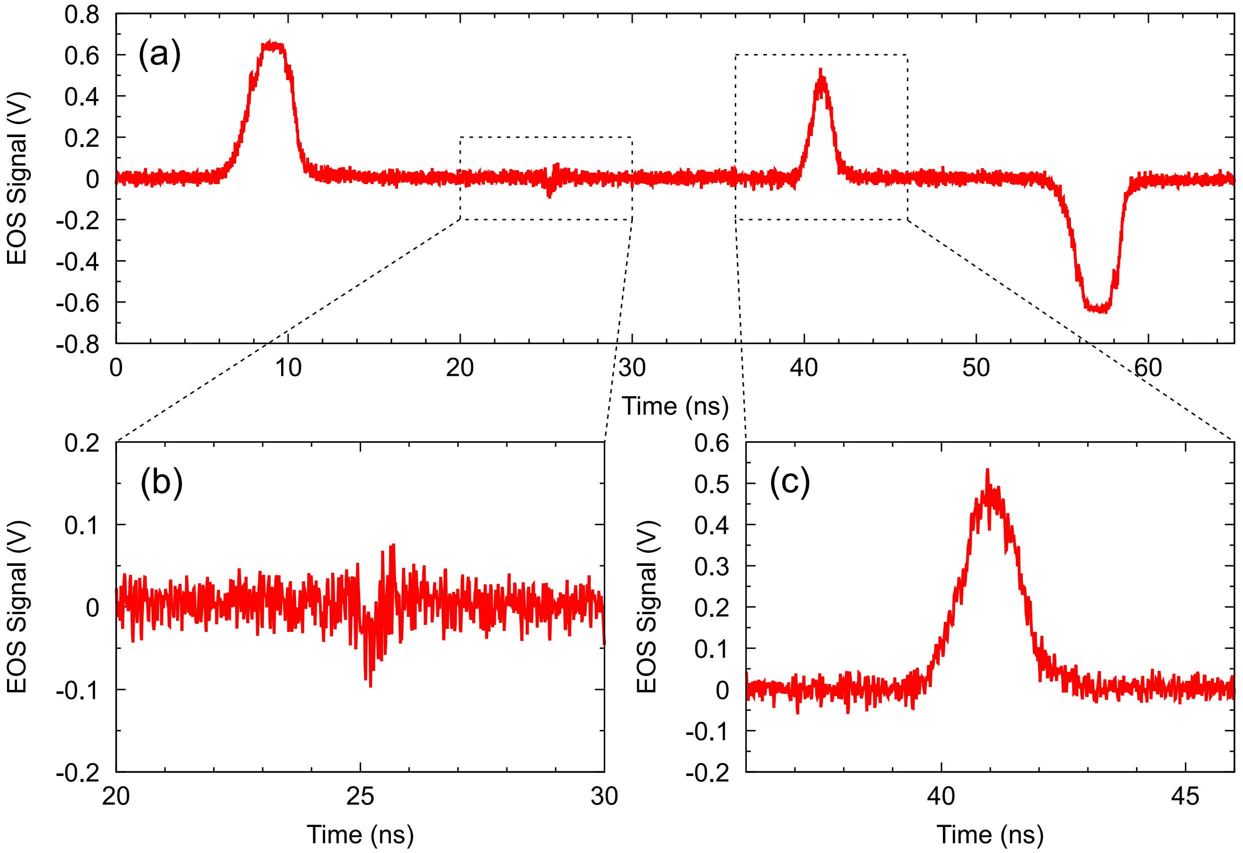

Figure 2. Typical signal at the balanced detector's output (oscilloscope trace). (a) Whole signal. (b) zoom of the background signal, i.e., without electric field. (c) zoom of the electro-optic signal. See supplementary Figure 1 for timing details.
